# Draft Genome Sequence of *Botrytis cinerea* BcDW1, Inoculum for Noble Rot of Grape Berries

**DOI:** 10.1128/genomeA.00252-13

**Published:** 2013-05-23

**Authors:** Barbara Blanco-Ulate, Greg Allen, Ann L. T. Powell, Dario Cantu

**Affiliations:** Department of Viticulture and Enology, University of California—Davis, Davis, California, USAa; Department of Plant Sciences, University of California—Davis, Davis, California, USAb; Dolce Winery, Oakville, California, USAc

## Abstract

Botrytized wines are produced from grape berries infected by *Botrytis cinerea* under specific environmental conditions. Here, we report the draft genome sequence of *B. cinerea* BcDW1, a strain isolated from Sémillon grapes in Napa Valley in 1992 that is used with the intent to induce noble rot for botrytized wine production.

## GENOME ANNOUNCEMENT

*Botrytis cinerea* (teleomorph, *Botryotinia fuckeliana*) is a necrotrophic plant pathogen that is particularly aggressive on ripe fruit (1). Under specific environmental conditions, infections of wine grape berries by *B. cinerea* cause noble rot, characterized by physicochemical modifications of infected berries that contribute to the unique properties of the botrytized wines (2). Changes attributed to noble rot include dehydration as result of the actions of fungal cell wall-degrading enzymes, oxidation of phenolic compounds by fungal laccases, and changes in the aroma profile caused by the breakdown of fruit esters and the synthesis of fungal volatiles (2–4).

*B. cinerea* isolate BcDW1 was recovered in 1992 from grape berries (*Vitis vinifera* cv. “Sémillon”) in Napa, California, and this isolate has been used as source of inoculum to induce noble rot in the production of Dolce Wine (Oakville, CA). DNA from an axenic BcDW1 culture was extracted using a modified cetyltrimethylammonium bromide (CTAB) method (5), and 6.9 Gb of Illumina HiSeq 2000 sequence reads was generated to achieve >100× coverage of the genome. After quality trimming (*Q* > 30), 99.78% of the reads were assembled into a nuclear genome composed of 453 scaffolds and a total length of 42.1 Mb (N_50_, 194 kb; L_50_, 69; gaps, 58 kb; G+C, 42%; median coverage, 125.3×). The complete mitochondrial genome was assembled into a single contig of 84 kb. *De novo* assembly was done using CLC Genomic Workbench v6.0 with the parameters optimized to achieve the highest completeness of the gene space estimated using Core Eukaryotic Genes Mapping Approach (CEGMA) (6). The BcDW1 genome was estimated to be >98% complete by mapping 248 low-copy core eukaryotic genes conserved across eukaryotes (6). Fifty-three million BcDW1 reads were uniquely aligned to the B05.10 (7) and T4 (8) scaffolds using Novoalign (v2.08.02, Novocraft), and 162,882 (4.0 single-nucleotide polymorphisms [SNPs]/kb) and 162,464 (3.9 SNPs/kb) SNP variants were identified by comparisons to the B05.10 and T4 genomes, respectively (Freebayes v0.9.9 [9]). A total of 5,620 nonsynonymous substitutions and 46 early stop codons were identified.

After masking repeats with RepeatMasker (10), gene models were identified with Augustus (11) using B05.10 transcripts (7), which were training sets for *ab initio* gene prediction and for evidence-based gene finding. As result, 11,073 complete gene models were identified. We found a large set of candidate secreted proteins (SignalP v4.0 [12]) that are involved in plant tissue penetration and decomposition, including 165 glycoside hydrolases, 44 carbohydrate esterases, and 10 polysaccharide lyases (CAZy database [13]). The most abundant CAZy families identified among these secreted proteins were 19 polygalacturonases (GH28), 15 xyloglucanases (GH16), 10 cutinases (CE5), and 9 pectin/pectate lyases (PL1 and PL3). We also detected other secreted proteins that we predict are relevant for noble rot, such as 3 laccases (2) and 9 carboxylesterases (14). The BcDW1 draft genome sequence will be useful for comparative studies as the genome sequences of more *B. cinerea* isolates become available, and it will contribute to elucidating the genetic bases of host specialization and the commercially relevant roles of *B. cinerea*.

### Nucleotide sequence accession numbers.

This Whole-Genome Shotgun project has been deposited at DDBJ/EMBL/GenBank under the accession no. AORW00000000. The version described in this paper is the first version, accession no. AORW01000000.
